# Dual contextual learning for semi-supervised medical image classification

**DOI:** 10.3389/fmed.2026.1810643

**Published:** 2026-05-20

**Authors:** Jiaying Liu, Chengyang Li, Sangsha Fang, Fangfang Deng, Qinghu He, Miao Cao

**Affiliations:** 1 Hunan University of Chinese Medicine, Changsha, China; 2 Central South University of Forestry and Technology, Changsha, China; 3 Wenzhou Business College, Wenzhou, China

**Keywords:** contrastive learning, image classification, image processing, medical image analysis, semi-supervised learning

## Abstract

Semi-supervised learning (SSL) has emerged as a promising paradigm for medical image classification, addressing the critical challenge of limited labeled data in healthcare where expert annotation is expensive and time-consuming. Existing pseudo-labeling methods generate labels for each unlabeled sample independently based on model confidence, which often proves unreliable when dealing with ambiguous pathological features in early-stage lesions or borderline cases, leading to error accumulation. However, medical images contain rich contextual information—samples with similar pathological characteristics naturally cluster in feature space, and maintaining consistency within these neighborhoods could provide more robust supervision. In this paper, we propose a Hierarchical Semantic Calibration (HSC) framework that leverages these contextual relationships to enhance pseudo-labeling reliability. We introduce two complementary modules: (1) a local semantic neighborhood alignment that enforces consistency among mutual k-nearest neighbors sharing similar pathological features, reducing isolated labeling errors through collective evidence; and (2) a global cluster-prototype calibration that aligns class-level representations across different augmented views through contrastive learning, ensuring disease categories maintain consistent patterns despite imaging variations. Additionally, we introduce a neighborhood-prototype consistency regularization that bridges these two scales, adaptively weighting the alignment between local neighborhoods and global prototypes based on neighborhood compactness, ensuring hierarchical consistency from local pathological features to global disease patterns. Extensive experiments demonstrate that HSC consistently outperforms state-of-the-art methods, achieving 92.24% accuracy on NCT-CRC-HE with only 200 labeled samples (2.97% improvement over PEFAT) and 94.17% on ISIC2018 with 20% labeled data (2.21% improvement).

## 1 Introduction

Deep learning has achieved remarkable success in various computer vision tasks due to the availability of large-scale annotated datasets (1, 2). However, while demonstrating outstanding performance in medical image analysis (MIA) (3–7), acquiring labeled data in this domain is substantially more costly as it requires annotation by medical professionals. This challenge has motivated the exploration of semi-supervised learning (SSL) approaches that can effectively utilize abundant unlabeled clinical data alongside limited labeled samples.

Semi-supervised learning has attracted significant attention for its ability to achieve strong performance using only a small amount of labeled data alongside a large volume of unlabeled data (8–10). Recent studies in SSL often explore two dominant paradigms: consistency-based regularization and pseudo-labeling strategies. Consistency learning, often combined with self-supervised pre-training, encourages stable predictions under perturbations and has demonstrated state-of-the-art results in SSL tasks (11–13). In contrast, pseudo-labeling methods assign labels to confidently predicted unlabeled samples and have shown strong potential (14–16). Among these approaches, PEFAT (17) achieves state-of-the-art performance by improving pseudo-label quality through Gaussian Mixture Models for loss distribution modeling and adversarial feature regularization.

**Figure 1:**
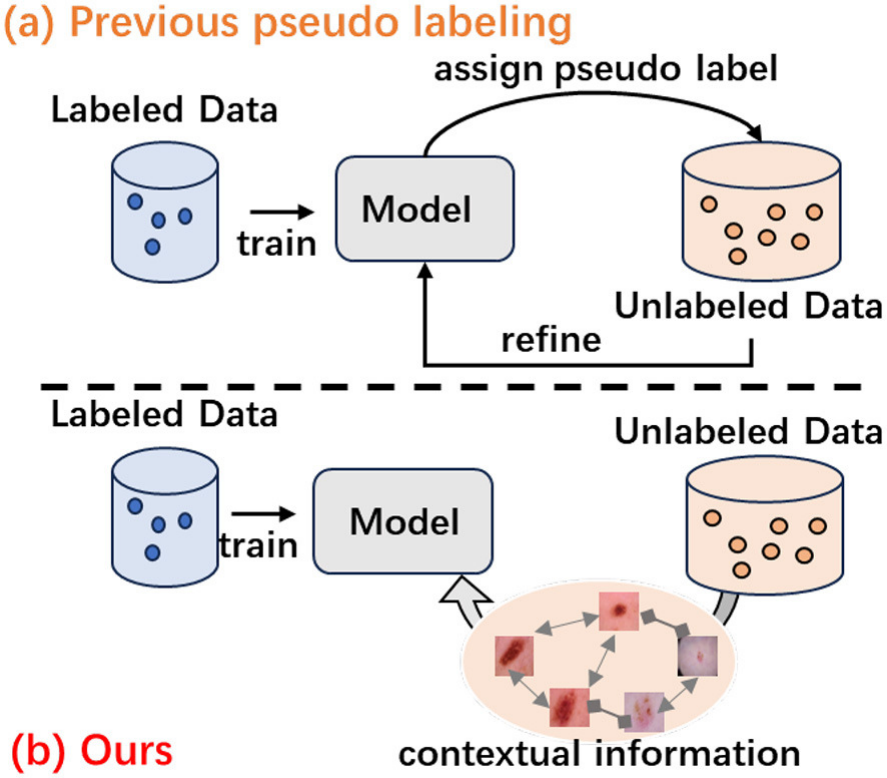
A brief comparison between previous pseudo labeling methods and our approach. **(a)** Pseudo labeling methods lead to cumulative bias. **(b)** Our method can extract unique contextual semantic information from medical images.

However, existing pseudo-labeling approaches including PEFAT generate labels for each sample independently. This instance-level approach proves problematic in medical imaging, where ambiguous pathological features in early-stage lesions or borderline cases often lead to unreliable predictions and error accumulation. Critically, medical images contain rich contextual information that remains unexploited—samples with similar pathological characteristics naturally cluster in feature space, forming semantic neighborhoods that could provide collective evidence for more reliable predictions. This observation motivates us to leverage contextual semantic information to enhance representation learning, as illustrated in Figure 1.

**Figure 2:**
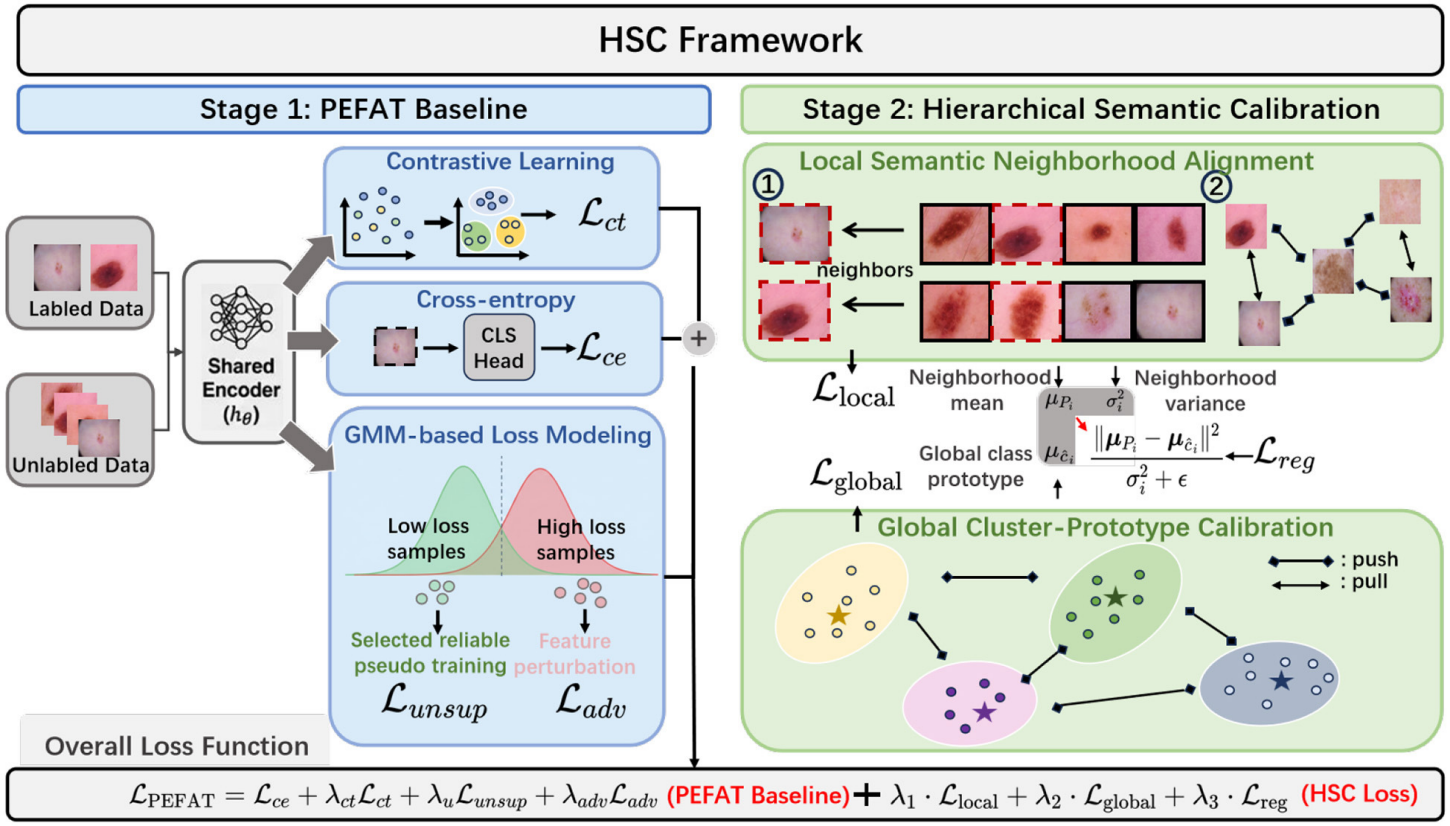
The architecture of our proposed framework, which integrates our Hierarchical Semantic Calibration (HSC) module into the PEFAT baseline. While PEFAT performs initial loss-based sample separation, our HSC module provides hierarchical supervision. It models semantic relationships at the local level and the global level, and finally bridges these two scales through Neighborhood-Prototype Consistency Regularization.

In this paper, we propose Hierarchical Semantic Calibration (HSC), the first framework to systematically exploit multi-level contextual relationships for semi-supervised medical image classification (Figure 2). Unlike existing methods that merely refine pseudo-labels, HSC introduces a novel dual-level supervision paradigm that fundamentally changes how unlabeled medical images contribute to learning. Building upon PEFAT's instance-level refinement, we introduce an orthogonal dimension of supervision through hierarchical semantic constraints. Our key innovation lies in recognizing that medical images possess rich contextual structures at multiple granularities—from local pathological similarities to global disease patterns—that can provide robust supervision signals beyond traditional pseudo-labels.

HSC introduces three synergistic components that form a hierarchical supervision system:

- **Local semantic neighborhood alignment:** This module identifies mutual *k*-nearest neighbors in feature space and enforces consistency among samples sharing similar pathological features, providing collective regularization that reduces isolated labeling errors particularly in ambiguous cases.
- **Global cluster-prototype calibration:** At the class level, we align prototypes computed from different augmented views through contrastive learning, ensuring disease categories maintain consistent representations despite imaging variations, providing robust class-level supervision.
- **Neighborhood-prototype consistency regularization:** This component bridges local and global scales by aligning neighborhood means with class prototypes, adaptively weighted by neighborhood compactness to handle samples with varying certainty levels.

The integration of these contextual mechanisms with PEFAT creates a powerful framework that addresses multiple challenges in semi-supervised medical image classification through multi-scale supervision at different granularities. The main contributions of this work are three-fold:

- We propose HSC, a framework that reconceptualizes semi-supervised medical image classification through dual-level semantic regularization—exploiting both local neighborhood and global cluster-level contextual relationships—further refined by an adaptive consistency regularization that bridges these two levels.
- We develop three synergistic modules: (i) local semantic alignment leveraging mutual k-nearest neighbors to enforce consistency among similar samples, mitigating pseudo-label errors through collective regularization; (ii) global prototype calibration maintaining class-level invariance across augmentations through contrastive learning; (iii) neighborhood-prototype consistency regularization that bridges and refines the dual-level supervision based on neighborhood compactness.
- Extensive experiments demonstrate that HSC significantly outperforms state-of-the-art methods across multiple benchmarks: 92.24% accuracy on NCT-CRC-HE (200 labels), 94.17% on ISIC2018 (20% labels), with notable 2.77% sensitivity improvement for minority classes—addressing the critical challenge of rare disease detection in clinical practice.

## 2 Related work

### 2.1 Semi-supervised learning

Semi-supervised learning (SSL) leverage both labeled and unlabeled data to enhance model performance. Representative approaches include entropy minimization (18–20), pseudo-labeling (also known as self-training) (21–24), and consistency regularization (25–28), all of which encourage the model to make confident and consistent predictions on unlabeled samples. For instance, Noise Student (24) leverages an iterative learning process in which pseudo-labels are generated by the continuously updated teacher model, enabling the student model to learn from all available data. Modern approaches like FixMatch (29) and FlexMatch (9) further refine pseudo-label quality through threshold mechanisms based on confidence scores.

### 2.2 Semi-supervised learning in medical image analysis

SSL has gained significant traction in medical image analysis due to the high cost and complexity of obtaining labeled medical data. It has been extensively applied in various tasks such as medical image detection (30, 31), classification (17, 32–35), and segmentation (36–38). For instance, FocalMix (30) introduces an SSL approach for 3D medical object detection by extending focal loss to enhance training stability. In Wang et al. (30), SSL is employed to effectively improve both disease grading and lesion segmentation performance. Recently, the ACPL (39) network is developed to refine pseudo-labels by generating them through informative mixup. However, previous representation learning in medical images have focused on instance-level supervised signals while ignoring the underlying group relationships in medical images, which can cause degradation of representation learning.

### 2.3 Contrastive learning

CL has emerged as a powerful paradigm for learning discriminative representations (40), primarily by encouraging similarity between positive pairs and dissimilarity between negative pairs. To boost its performance, several methods have integrated label information into contrastive frameworks (16, 41, 42). An influential line of work is prototypical contrastive learning (43), which leverages *k*-means clustering across the entire dataset to construct class-level prototypes for guiding high-level representation learning. Nonetheless, the periodic re-assignment of pseudo-labels (typically once per epoch) introduces substantial computational overhead. To address this, IDFD (44) improves upon the prototypical framework by introducing feature decorrelation mechanisms. Additionally, ProPos (45) proposes a prototype scattering loss that updates prototypes dynamically within each mini-batch, while still relying on epoch-level pseudo-label updates, thereby further enhancing the quality of learned features.

## 3 Methodology

In this section, we present our Hierarchical Semantic Calibration (HSC) framework (Figure 2) for semi-supervised medical image classification. We first formulate the problem setting (Section 3.1) and review our baseline PEFAT method (Section 3.2). We then introduce our dual-level semantic calibration approach (Section 3.3), consisting of local semantic neighborhood alignment and global cluster-prototype calibration, along with a consistency regularization that bridges these two levels. Finally, we describe the overall training objective that integrates these components (Section 3.4). The key insight of our approach is to move beyond instance-level pseudo-labels by exploiting the inherent hierarchical semantic structure of medical images through complementary local and global regularization, unified by adaptive cross-level consistency constraints. The overall process is shown in pseudocode (Algorithm 1).

Algorithm 1 Hierarchical semantic calibration (HSC). 
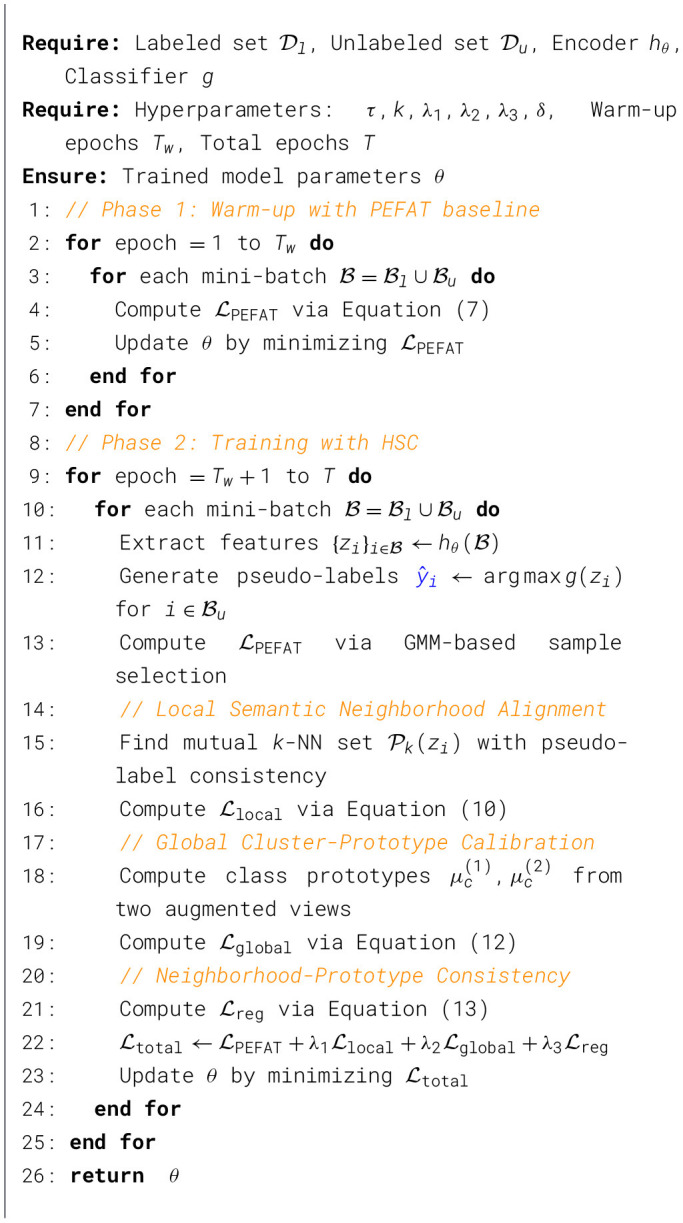

### 3.1 Problem formulation

In the semi-supervised classification task for medical imaging, we are given a small labeled dataset $D_{l}={\left\{\left(x_{i},y_{i}\right)\right\}}_{i=1}^{N_{l}}$ and a much larger unlabeled dataset $D_{u}={\left\{u_{j}\right\}}_{j=1}^{N_{u}}$, where *N*_*l*_ ≪ *N*_*u*_. It is typically assumed that both labeled and unlabeled samples are drawn from the same underlying data distribution. The objective of semi-supervised learning in this context is to leverage the abundant unlabeled medical data $D_{u}$ to improve the performance of classification models trained with the limited labeled set $D_{l}$. Different methods mainly vary in how they exploit $D_{u}$ for representation learning and label inference.

### 3.2 Baseline method: PEFAT

Our approach leverages PEFAT (17) as the foundational framework. PEFAT is based on the **dividable pseudo-loss assumption**—clean samples exhibit lower training losses while noisy samples show higher losses—and employs GMM-based loss modeling, cross-augmentation, and adversarial feature regularization to discriminate between reliable and unreliable pseudo-labels in semi-supervised medical image classification.

#### 3.2.1 Discriminative feature learning via contrastive objective

PEFAT incorporates contrastive learning to extract discriminative representations. For a batch containing labeled samples $B_{l}$ and unlabeled samples $B_{u}$, the self-supervised contrastive objective is formulated as:


$$\begin{array}{l} L_{ct}=-\frac{1}{2|B|}\overset{2|B|}{\underset{i=1}{\sum }}\log\frac{\exp\left(v_{i}\cdot v_{i}^{+}/\tau \right)}{\sum_{j=1}^{2|B|}{⊮}_{\left(j\neq i\right)}\exp\left(v_{i}\cdot v_{j}/\tau \right)}, \end{array}$$ (1)

where $\left|B|=|B_{l}|+|B_{u}\right|$ indicates batch size, *v*_*i*_ and *v*_*j*_ are unit-normalized embeddings, $v_{i}^{+}$ represents the positive augmented counterpart, and τ is the temperature coefficient. This objective (Equation 1) facilitates learning transformation-invariant yet semantically discriminative features.

#### 3.2.2 Gaussian mixture modeling for loss characterization

Based on the dividable pseudo-loss assumption, the framework employs GMM to model loss distributions on labeled data $D_{l}$. The sample-wise loss collection (Equation 2) is defined as:


$$\begin{array}{l} L\left(D_{l}|f_{\theta }\right)=\left\{-y_{i}\log\left(f_{\theta }\left({ŷ}_{i}|x_{i}\right)\right),x_{i}\in D_{l}\right\}, \end{array}$$ (2)

where *f*_θ_ denotes the network and ŷ_*i*_ its output. The mixture model's density function takes the form:


$$\begin{array}{l} G\left(\ell_{i}\right)=\overset{K-1}{\underset{k=0}{\sum }}\alpha_{k}G_{k}\left(\ell_{i}|m_{k},S_{k}\right),\;\ell_{i}\in L\left(D_{l}|f_{\theta }\right), \end{array}$$ (3)

with mixture weights α_*k*_ satisfying $\overset{K-1}{\underset{k=0}{\sum }}\alpha_{k}=1$, and (*m*_*k*_, *S*_*k*_) representing component-specific parameters optimized via the EM algorithm. This probabilistic modeling (Equation 3) facilitates principled separation of clean and noisy pseudo-labels based on loss statistics.

#### 3.2.3 Cross-augmentation for robust pseudo-loss estimation

To address model overconfidence and improve pseudo-loss estimation, PEFAT employs bidirectional cross-view supervision. For unlabeled data $u_{i}\in D_{u}$, two augmentation branches produce mutual supervision signals (Equation 4):


$$\begin{array}{l} p_{i}^{1}=h_{\theta }\left(A_{s1}\left(u_{i}\right)\right),\;p_{i}^{2}=h_{\theta }\left(A_{s2}\left(u_{i}\right)\right), \end{array}$$ (4)

where $A_{s1}$ applies geometric transformations (affine, rotation, and cutout) while $A_{s2}$ performs appearance alterations (grayscale, color distortion, blur). The cross-supervision loss (Equation 5) becomes:


$$\begin{array}{l} \ell_{i}^{cross}=\frac{1}{2}\left[L_{CE}\left(p_{i}^{1},\text{sg}\left(p_{i}^{2}\right)\right)+L_{CE}\left(p_{i}^{2},\text{sg}\left(p_{i}^{1}\right)\right)\right], \end{array}$$ (5)

where sg(·) denotes stop-gradient operation to prevent collapse.

#### 3.2.4 Sample selection and adversarial feature regularization

Based on GMM-fitted distributions and cross-view losses, PEFAT selects reliable pseudo-labeled instances to form ${\tilde{D}}_{u}$ with low loss values. For high-loss samples potentially containing noisy labels, instead of discarding them, PEFAT applies feature-level adversarial perturbations (Equation 6):


$$\begin{array}{l} r_{adv}=\epsilon \cdot \frac{\nabla_{z}L_{CE}\left({ŷ}_{i},g\left(z\right)\right)}{||\nabla_{z}L_{CE}\left({ŷ}_{i},g\left(z\right)\right)|{|}_{2}}, \end{array}$$ (6)

with *z* = *h*(*x*_*i*_) being the feature encoding, *g*(·) the classifier, and ϵ the perturbation radius. This regularization smooths the decision boundary and extracts discriminative information from uncertain samples.

#### 3.2.5 Unified training loss

The complete training objective (Equation 7) aggregates all loss terms:


$$\begin{array}{l} \begin{array}{cc} L_{\text{PEFAT}}=\; & L_{ce}+\lambda_{ct}L_{ct}+\lambda_{u}L_{unsup}+\lambda_{adv}L_{adv}, \end{array} \end{array}$$ (7)

where $L_{ce}$ denotes the supervised cross-entropy loss computed on weakly-augmented labeled samples. is the supervised objective on weakly-augmented labeled samples, $L_{unsup}$ represents pseudo-supervision on selected samples, $L_{adv}$ provides adversarial regularization, with λ_*ct*_, λ_*u*_, λ_*adv*_ as weighting factors.

### 3.3 Hierarchical semantic calibration

Building upon PEFAT's instance-level refinement, we propose Hierarchical Semantic Calibration (HSC) that explicitly exploits the contextual relationships inherent in medical images. Unlike PEFAT which treats each sample independently, HSC introduces hierarchical semantic regularization through three synergistic components:

#### 3.3.1 Local semantic neighborhood alignment

To capture fine-grained semantic relationships beyond instance-level pseudo-labels, we introduce a local semantic alignment mechanism that enforces consistency among mutually similar samples in the feature space. The key insight is that mutually-similar samples within the top-*k* nearest neighbors that share the same pseudo-label should maintain consistent representations, thereby providing robust regularization against noisy predictions.

For a mini-batch B with feature representations ${\left\{z_{i}\right\}}_{i\in B}$ extracted by the encoder, we first identify the mutual *k*-nearest neighbors (Equation 8) for each sample:


$$\begin{array}{l} M_{k}\left(z_{i}\right)=\left\{z_{j}\in B|z_{j}\in N_{k}\left(z_{i}\right)\wedge z_{i}\in N_{k}\left(z_{j}\right)\right\}, \end{array}$$ (8)

where $N_{k}\left(z_{i}\right)$ denotes the top-*k* nearest neighbors of *z*_*i*_ based on cosine similarity in the feature space. The mutual neighborhood constraint ensures bidirectional similarity, providing more reliable semantic relationships than unidirectional neighbors.

Subsequently, we define the semantic neighbors (Equation 9) by incorporating pseudo-label information:


$$\begin{array}{l} P_{k}\left(z_{i}\right)=\left\{z_{j}|z_{j}\in M_{k}\left(z_{i}\right)\text{and}{ŷ}_{i}={ŷ}_{j}\right\}, \end{array}$$ (9)

where ŷ_*i*_ and ŷ_*j*_ represent the pseudo-labels of samples *i* and *j*, respectively. Samples in $P_{k}\left(z_{i}\right)$ are considered semantically aligned with *z*_*i*_, as they exhibit both feature proximity and label agreement.

Based on these semantic neighborhoods, we formulate the local alignment loss (Equation 10):


$$\begin{array}{l} L_{\text{local}}=\frac{1}{\left|B{|}^{2}-|B\right|}\underset{z_{i}\in B,z_{j}\in B,i\neq j}{\sum }s_{ij}\cdot d_{ij}+\left(1-s_{ij}\right)\cdot \left(\delta -d_{ij}\right), \end{array}$$ (10)

where *s*_*ij*_ = 1 if $z_{j}\in P_{k}\left(z_{i}\right)$ and *s*_*ij*_ = 0 otherwise, *d*_*ij*_ = 1 − cos(*z*_*i*_, *z*_*j*_) is the cosine distance, and δ is a margin parameter.

#### 3.3.2 Global cluster-prototype calibration

While local neighborhood alignment captures fine-grained semantic relationships, we further introduce a global calibration mechanism that maintains consistency at the cluster level. This module leverages class prototypes to ensure that the learned representations respect the underlying categorical structure of medical images across different augmented views.

For each pseudo-class *c*∈{1, ..., *C*} in the mini-batch B, we compute prototypes from two different augmented views of the same samples. Let $z_{i}^{\left(1\right)}$ and $z_{i}^{\left(2\right)}$ denote the feature representations of sample *i* under two different augmentations. The prototypes are computed as:


$$\begin{array}{l} \mu_{c}^{\left(1\right)}=\frac{1}{\left|G_{c}\right|}\underset{i\in G_{c}}{\sum }z_{i}^{\left(1\right)},\;\mu_{c}^{\left(2\right)}=\frac{1}{\left|G_{c}\right|}\underset{i\in G_{c}}{\sum }z_{i}^{\left(2\right)} \end{array}$$ (11)

where $G_{c}=\left\{i\in B|\arg\max\left({ŷ}_{i}\right)=c\right\}$ represents the set of samples assigned to pseudo-class *c*.

To ensure cross-view consistency at the prototype level, we employ a contrastive loss that pulls together prototypes of the same class across different views while pushing apart prototypes of different classes:


$$\begin{array}{l} L_{\text{global}}=-\frac{1}{C}\overset{C}{\underset{c=1}{\sum }}\log\frac{\exp\left(\cos\left(\mu_{c}^{\left(1\right)},\mu_{c}^{\left(2\right)}\right)/\tau_{u}\right)}{\sum_{j=1}^{C}\exp\left(\cos\left(\mu_{c}^{\left(1\right)},\mu_{j}^{\left(2\right)}\right)/\tau_{u}\right)}, \end{array}$$ (12)

where cos(·, ·) denotes cosine similarity and τ_*u*_ is a temperature parameter that controls the sharpness of the distribution.

This global calibration provides coarse-grained supervision that complements the fine-grained local alignment. By maintaining prototype consistency across augmentations, the model learns view-invariant categorical representations that are robust to data variations commonly encountered in medical imaging, such as differences in imaging protocols, scanner settings, or patient positioning.

#### 3.3.3 Neighborhood-prototype consistency regularization

To strengthen the connection between our local and global calibration mechanisms, we introduce a consistency regularization that aligns the local neighborhood structure with global prototypes. Specifically, we encourage each sample's local neighborhood mean to be consistent with its corresponding class prototype, weighted by the neighborhood compactness:


$$\begin{array}{l} L_{\text{reg}}=\frac{1}{\left|B\right|}\underset{i\in B}{\sum }\frac{||\mu_{P_{i}}-\mu_{{ĉ}_{i}}|{|}^{2}}{\sigma_{i}^{2}+\epsilon }, \end{array}$$ (13)

where $\mu_{P_{i}}=\frac{1}{\left|P_{k}\left({\text{z}}_{i}\right)\right|}\underset{j\in P_{k}\left({\text{z}}_{i}\right)}{\sum }{\text{z}}_{j}$ is the local neighborhood mean, **μ**_ĉ_*i*__ is the global prototype, and $\sigma_{i}^{2}=\frac{1}{\left|P_{k}\left({\text{z}}_{i}\right)\right|}\underset{j\in P_{k}\left({\text{z}}_{i}\right)}{\sum }||{\text{z}}_{i}-{\text{z}}_{j}|{|}^{2}$ measures neighborhood variance. This term creates a bridge between local and global scales—samples with compact neighborhoods (small $\sigma_{i}^{2}$) are enforced to align strongly with their class prototypes, while boundary samples with dispersed neighborhoods receive adaptive regularization. This hierarchical consistency is particularly valuable for medical images where local pathological features should align with global disease patterns.

### 3.4 Overall training objective

Our Hierarchical Semantic Calibration framework integrates the baseline PEFAT loss with the proposed dual-level semantic regularization:


$$\begin{array}{l} L_{\text{total}}=L_{\text{PEFAT}}+\lambda_{1}\cdot L_{\text{local}}+\lambda_{2}\cdot L_{\text{global}}+\lambda_{3}\cdot L_{\text{reg}} \end{array}$$ (14)

where λ_1_, λ_2_, and λ_3_ are weighting coefficients that balance the contribution of local and global calibration losses. λ_3_ controls the regulation term.

## 4 Experiments

### 4.1 Datasets and implementation details

#### 4.1.1 Datasets

We evaluate our proposed HSC framework on two widely-used medical image classification benchmarks that cover different imaging modalities and diagnostic tasks: **NCT-CRC-HE-100K** (46): 100,000 histopathological image patches of colorectal cancer tissue, 9 tissue classes, 70/10/20 train/val/test split. **ISIC2018** (47): 10,015 dermoscopic images across 7 disease categories with significant class imbalance. Following recent state-of-the-art protocols (35), the datasets are split into 70%/10%/20% for training/validation/test systematically in each fold.

#### 4.1.2 Implementation details

We follow the experimental configuration of PEFAT (17), employing DenseNet-121 (48) as the backbone (encoder) with 224 × 224 input resolution. Training utilizes Adam optimizer with learning rate 0.001, batch size of 16 labeled and 48 unlabeled samples, and 80 total epochs (30 warm-up, 50 training). Our method was implemented based on Pytorch, using two NVIDIA Gefore RTX 4090 GPUs. We retain PEFAT's hyperparameters (τ = 0.05, ϵ = 1, η = 0.5) and set our method-specific weights as λ_1_ = 0.5 for local semantic alignment, λ_2_ = 0.3 for global prototype calibration, and *k* = 10 for the mutual *k*-nearest neighbor search in local neighborhood alignment. The weight λ_3_ for the regular term $L_{\text{reg}}$ is set to 0.1. The margin δ in Equation 10 is set to 1.0 and the temperature value τ_*u*_ in HSC phase is set to 0.1.

### 4.2 Evaluation metrics

To quantitatively evaluate the performance of our proposed method, five widely-adopted metrics are employed: Accuracy (ACC), Sensitivity (SENS), Specificity (SPEC), Precision (PREC), and F1-score (F1). These metrics are defined based on the components of the confusion matrix, including True Positives (*TP*), True Negatives (*TN*), False Positives (*FP*), and False Negatives (*FN*). The specific formulations are as follows:

- **ACC** measures the overall proportion of correctly classified pixels:
$$\begin{array}{l} \text{ACC}=\frac{TP+TN}{TP+TN+FP+FN} \end{array}$$ (15)
- **SENS** (also known as Recall) quantifies the ability of the model to correctly identify positive samples:
$$\begin{array}{l} \text{SENS}=\frac{TP}{TP+FN} \end{array}$$ (16)
- **SPEC** reflects the model's capability to exclude negative samples (e.g., background):
$$\begin{array}{l} \text{SPEC}=\frac{TN}{TN+FP} \end{array}$$ (17)
- **PREC** evaluates the proportion of true positive predictions among all positive predictions made by the model:
$$\begin{array}{l} \text{PREC}=\frac{TP}{TP+FP} \end{array}$$ (18)
- **F1** is the harmonic mean of Precision and Recall, providing a balanced measurement of the segmentation quality:
$$\begin{array}{l} \text{F1}=\frac{2\cdot TP}{2\cdot TP+FP+FN} \end{array}$$ (19)

### 4.3 Comparisons

#### 4.3.1 Results on the NCT-CRC-HE

Table 1 presents comprehensive evaluation results on the NCT-CRC-HE dataset under two label-scarce scenarios (100 and 200 labeled data). Our HSC framework demonstrates substantial improvements over the PEFAT baseline and achieves state-of-the-art performance across all metrics. With 200 labeled samples, our method attains **92.24%** ACC, representing a **2.97%** improvement over PEFAT (**89.27%**). Moreover, our approach outperforms the previous best method ReCo by **0.73%** and substantially exceeds traditional SSL methods like FixMatch (**82.62%**) and CoMatch (**85.72%**). In the extremely limited 100-label setting, our method reaches **87.41%** accuracy—a **1.64%** gain over PEFAT (**85.77%**).

**Table 1:** Results from 5-fold cross-validation (mean ± std) on the **NCT-CRC-HE** dataset using 100 and 200 labeled samples.

Method	NCT-CRC-HE (200 labeled data)				NCT-CRC-HE (100 labeled data)			
	ACC	SENS	PREC	F1	ACC	SENS	PREC	F1
MT (49)	80.87 ± 0.92	80.36 ± 1.21	82.67 ± 0.83	80.45 ± 0.87	76.28 ± 0.94	75.25 ± 0.70	77.68 ± 1.16	76.92 ± 0.61
FixMatch (8)	82.62 ± 0.57	83.96 ± 0.66	83.18 ± 0.71	83.28 ± 0.97	78.29 ± 1.23	78.75 ± 0.94	80.68 ± 0.46	79.80 ± 0.42
SimPLE (14)	83.95 ± 0.97	84.26 ± 0.77	84.03 ± 0.92	84.72 ± 0.32	80.71 ± 0.81	79.98 ± 1.16	81.78 ± 0.80	81.17 ± 0.72
CoMatch (50)	85.72 ± 0.56	86.06 ± 0.73	87.73 ± 0.25	85.58 ± 0.53	82.86 ± 0.79	83.27 ± 0.81	83.47 ± 0.43	83.92 ± 0.55
SimMatch (51)	87.68 ± 0.53	86.78 ± 0.79	88.21 ± 0.28	87.37 ± 0.42	83.16 ± 0.71	83.91 ± 0.73	83.21 ± 0.50	84.01 ± 0.36
RAC-MT (52)	86.06 ± 0.55	86.27 ± 0.51	88.11 ± 0.54	86.29 ± 0.30	82.17 ± 0.98	82.91 ± 0.74	82.50 ± 0.62	83.27 ± 0.81
ACPL (39)	88.12 ± 0.61	87.52 ± 0.46	89.82 ± 1.12	87.65 ± 0.52	83.34 ± 0.89	84.06 ± 0.68	85.32 ± 0.53	84.70 ± 0.51
PEFAT (17)	89.27 ± 0.58	88.92 ± 0.37	89.76 ± 0.89	89.53 ± 0.66	85.77 ± 0.82	84.98 ± 0.79	85.76 ± 0.47	85.15 ± 0.68
ReCo (35)	91.51 ± 0.47	90.96 ± 0.56	91.93 ± 0.37	91.22 ± 0.38	**87.56** **±** **0.73**	86.17 ± 0.56	87.57 ± 0.32	86.72 ± 0.35
Ours	**92.24** **±** **0.62**	**91.31** **±** **0.62**	**92.65** **±** **0.64**	**91.43** **±** **0.21**	87.41 ± 0.62	**88.02** **±** **0.74**	**88.31** **±** **0.51**	**87.28** **±** **0.24**

Bold and underlined values indicate the highest and second-highest performance, respectively.

#### 4.3.2 Results on the ISIC2018

Table 2 demonstrates our method's effectiveness on the ISIC2018 skin lesion classification task. With 20% labeled data, our approach achieves **94.17%** ACC, improving upon the PEFAT baseline by **2.21%** (from **91.96%**). This improvement extends to sensitivity, increasing from **74.43%** to **77.20%** (a **2.77%** gain). Our method also surpasses ReCo (**93.25%**) by **0.92%**. Under the extreme 5% label regime, our method maintains **91.24%** accuracy compared to PEFAT's **89.92%**, a **1.32%** improvement, with the F1 score increasing from **48.86%** to **52.01%** (**3.15%** gain).

**Table 2:** Results from 5-fold cross-validation (mean ± std) on the ISIC 2018 dataset using 5% and 20% labeled samples.

Method	ISIC2018 (20% labeled data)				ISIC2018 (5% labeled data)			
	ACC	SENS	SPEC	F1	ACC	SENS	SPEC	F1
SRC-MT (11)	90.31 ± 0.73	70.36 ± 0.91	90.39 ± 0.87	57.39 ± 0.72	86.72 ± 0.77	60.15 ± 0.97	88.58 ± 0.74	45.15 ± 0.63
DS^3^L (53)	89.72 ± 0.91	68.78 ± 0.74	90.06 ± 1.28	58.79 ± 0.86	84.72 ± 0.80	56.47 ± 0.79	88.03 ± 0.59	42.32 ± 0.86
FixMatch (8)	90.14 ± 0.55	69.79 ± 0.68	90.21 ± 0.73	57.83 ± 0.47	85.72 ± 0.46	57.75 ± 0.64	88.38 ± 0.86	44.81 ± 0.87
SimMatch (51)	91.16 ± 0.56	72.77 ± 0.63	91.65 ± 0.71	61.80 ± 0.58	88.30 ± 0.82	61.03 ± 0.74	89.52 ± 0.91	47.18 ± 0.80
CoMatch (50)	90.78 ± 0.62	71.60 ± 0.82	91.02 ± 0.66	60.39 ± 0.76	87.15 ± 0.71	60.67 ± 0.51	89.06 ± 0.77	46.75 ± 0.69
RAC-MT (52)	91.37 ± 0.71	73.57 ± 0.90	91.55 ± 0.40	62.10 ± 0.73	88.96 ± 0.50	61.92 ± 0.86	89.71 ± 0.82	47.90 ± 0.57
ACPL (39)	91.52 ± 0.64	74.07 ± 0.82	91.55 ± 0.64	64.68 ± 0.92	89.20 ± 0.58	62.32 ± 1.12	89.94 ± 0.64	48.60 ± 0.74
PEFAT (17)	91.96 ± 0.56	74.43 ± 0.72	91.70 ± 0.38	64.83 ± 0.68	89.92 ± 0.74	62.29 ± 0.53	90.02 ± 0.68	48.86 ± 0.82
ReCo (35)	93.25 ± 0.61	76.55 ± 0.89	**93.13** **±** **0.19**	66.07 ± 0.52	91.10 ± 0.60	**64.21** **±** **0.74**	91.31 ± 0.51	50.73 ± 0.38
**Ours**	**94.17** **±** **0.55**	**77.20** **±** **0.42**	93.07 ± 0.21	**66.41** **±** **0.70**	**91.24** **±** **0.32**	64.16 ± 0.55	**93.42** **±** **0.32**	**52.01** **±** **0.78**

Bold and underlined values indicate the highest and second-highest performance, respectively.

### 4.4 Ablation study

As shown in Table 3, the ablation results demonstrate the complementary nature of our proposed modules. Adding $L_{\text{local}}$ alone yields modest improvements(**0.91%** in ACC, **0.55%** in F1), indicating that local semantic alignment helps maintain feature consistency among similar samples. In contrast, $L_{\text{global}}$ alone provides stronger gains (**0.72%** in ACC, **0.41%** in F1), suggesting that prototype-level regularization is particularly effective for the class-imbalanced ISIC2018 dataset. The regularization term $L_{\text{reg}}$ bridges local and global constraints by adaptively weighting their alignment based on neighborhood compactness. When all components are combined, our full model achieves **2.21%** accuracy improvement, exceeding the sum of individual gains, which validates the synergistic relationship between local and global calibration mediated by the adaptive regularization. The substantial sensitivity improvement (+**2.77%**) indicates that our hierarchical approach is especially beneficial for detecting minority classes-a critical requirement in medical image analysis. This confirms that local fine-grained alignment, global cluster-prototype calibration, and their adaptive bridging provide mutually reinforcing supervision signals that enhance PEFAT's pseudo-loss estimation mechanism.

**Table 3:** Ablation study on ISIC2018 with 20% labeled samples.

Components			Performance metrics			
$L_{\text{global}}$	$L_{\text{local}}$	$L_{\text{reg}}$	**ACC**	**SENS**	**SPEC**	**F1**
✗	✗	✗	91.96	74.43	91.70	64.83
✓ ✗	✗ ✓	✗ ✗	92.68 92.87	75.32 75.21	92.15 92.18	65.24 65.38
✓ ✓ ✗	✓ ✗ ✓	✗ ✓ ✓	93.52 93.28 93.35	76.15 75.87 75.94	92.73 92.51 92.58	65.89 65.66 65.73
✓	✓	✓	**94.17**	**77.20**	**93.07**	**66.41**
*Improvement*			(+2.21)	(+2.77)	(+1.37)	(+1.58)

#### 4.4.1 Ablation on warm-up necessity

Following PEFAT, we adopt an initial warm-up strategy. As Table 4 shows, training HSC from scratch without it significantly degrades performance, confirming its necessity for establishing preliminary discriminative representations. Since our HSC relies heavily on feature similarities, computing mutual *k*-NN neighbors and global prototypes on chaotic, un-warmed features inevitably aligns semantically mismatched samples, triggering severe confirmation bias and error accumulation. Therefore, the warm-up serves as a vital prerequisite, providing a stable initial feature space for our dual-level calibration.

**Table 4:** Ablation study on the effect of the contrastive warm-up stage.

Method	ACC (%)	F1 (%)
HSC (w/o warm-up)	92.25	63.83
**HSC (w/warm-up)**	**94.17**	**66.41**

### 4.5 Hyperparameter analysis

**Figure 3:**
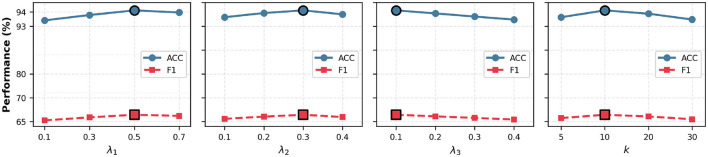
Hyperparameter sensitivity analysis on ISIC2018 (20% labeled). Impact of λ_1_, λ_2_, λ_3_ and *k* on ACC and F1 score. Optimal values: λ_1_ = 0.5, λ_2_ = 0.3, λ_3_ = 0.1, *k* = 10.

Figure 3 shows the sensitivity analysis of three key hyperparameters. The local alignment weight λ_1_ achieves optimal performance at 0.5, with accuracy dropping **1.05%** when λ_1_ < 0.3 due to insufficient regularization. The global calibration weight λ_2_ peaks at 0.3, balancing class-level consistency without over-rigidity. The bridging regularization weight λ_3_ performs best at 0.1, with performance gradually declining as it increases, suggesting that subtle adaptive weighting is more effective than aggressive enforcement. For neighborhood size *k*, the value of 10 provides the best trade-off—smaller values limit regularization while larger values (*k* ≥ 20) introduce noise, reducing accuracy to **93.21%** at *k* = 30. Notably, F1 scores show greater sensitivity than accuracy across all parameters, reflecting the challenge of imbalanced medical image classification. Table 5 examines the margin parameter δ in $L_{\text{local}}$. The optimal value δ = 1.0 achieves **91.24%** accuracy and **52.01%** F1 score. Smaller margins (δ = 0.5) reduce performance to **90.58%** ACC due to insufficient separation, while larger values (δ = 5.0) degrade to **88.67%** ACC by creating excessive gaps that hinder feature learning.

**Table 5:** Impact of margin parameter δ on ISIC2018 (5% labeled data).

Margin	ACC	SENS	SPEC	F1
δ = 0.5	90.58	62.45	92.76	50.34
δ = 1.0	**91.24**	**64.16**	**93.42**	**52.01**
δ = 2.0	90.82	63.28	93.05	51.12
δ = 5.0	88.67	60.89	91.45	49.78

Best results are in **bold**.

### 4.6 Visualization

#### 4.6.1 Error case analysis

**Figure 4:**
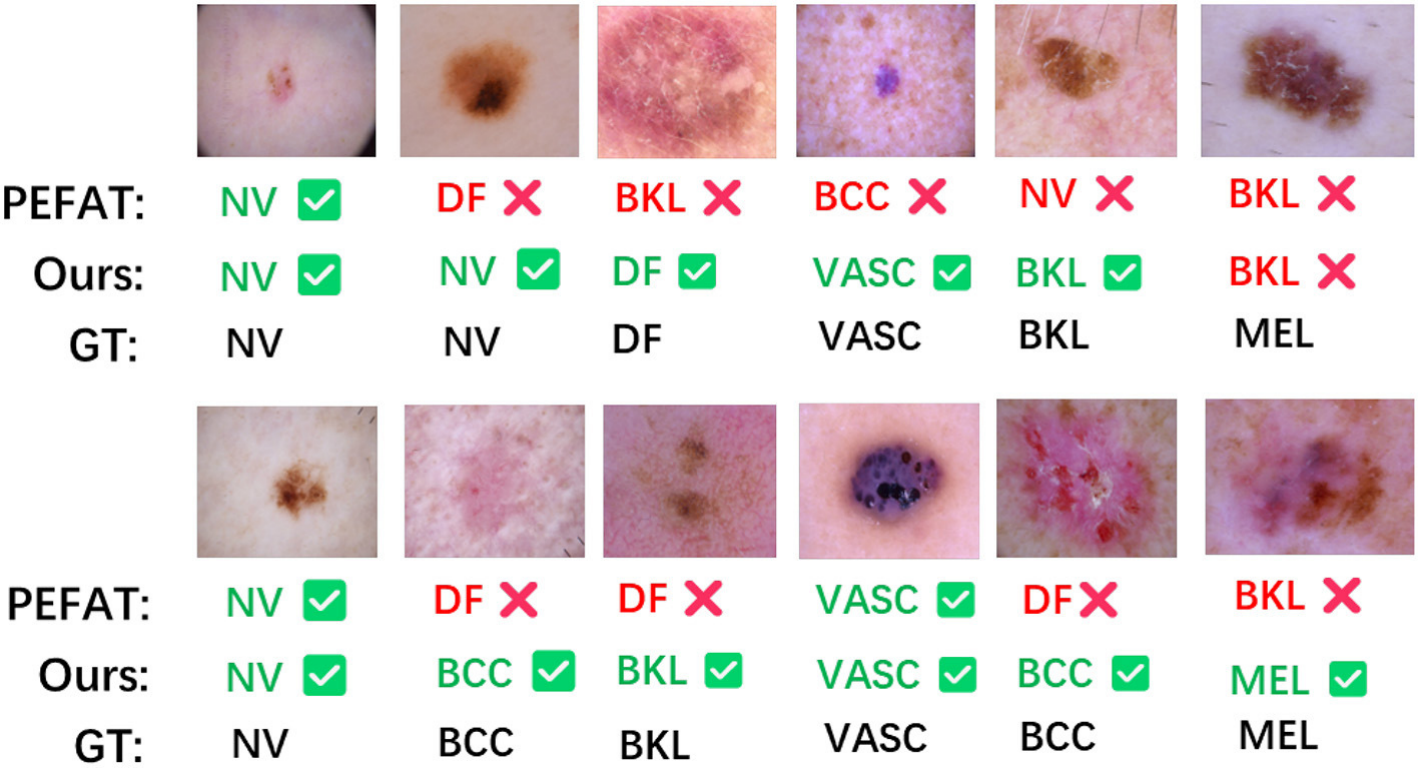
Error case analysis of our method and PEFAT on ISIC2018 (5% labeled data). Predicted labels are shown with 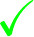 (correct) or 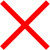 (incorrect). Ground truth (GT) labels at bottom.

Figure 4 presents representative failure modes in semi-supervised skin lesion classification. PEFAT exhibits a systematic bias toward frequent classes (NV and BKL), misclassifying 9 out of 12 samples including rare categories like VASC and DF. In contrast, HSC's hierarchical calibration effectively reduces this bias, correctly identifying visually similar lesions through better feature alignment. The rightmost case—a melanoma with atypical presentation—remains challenging for both methods, highlighting the inherent difficulty of MEL detection under limited supervision. This qualitative analysis corroborates our quantitative results, demonstrating HSC's improved robustness particularly for underrepresented classes.

#### 4.6.2 Feature visualization

**Figure 5:**
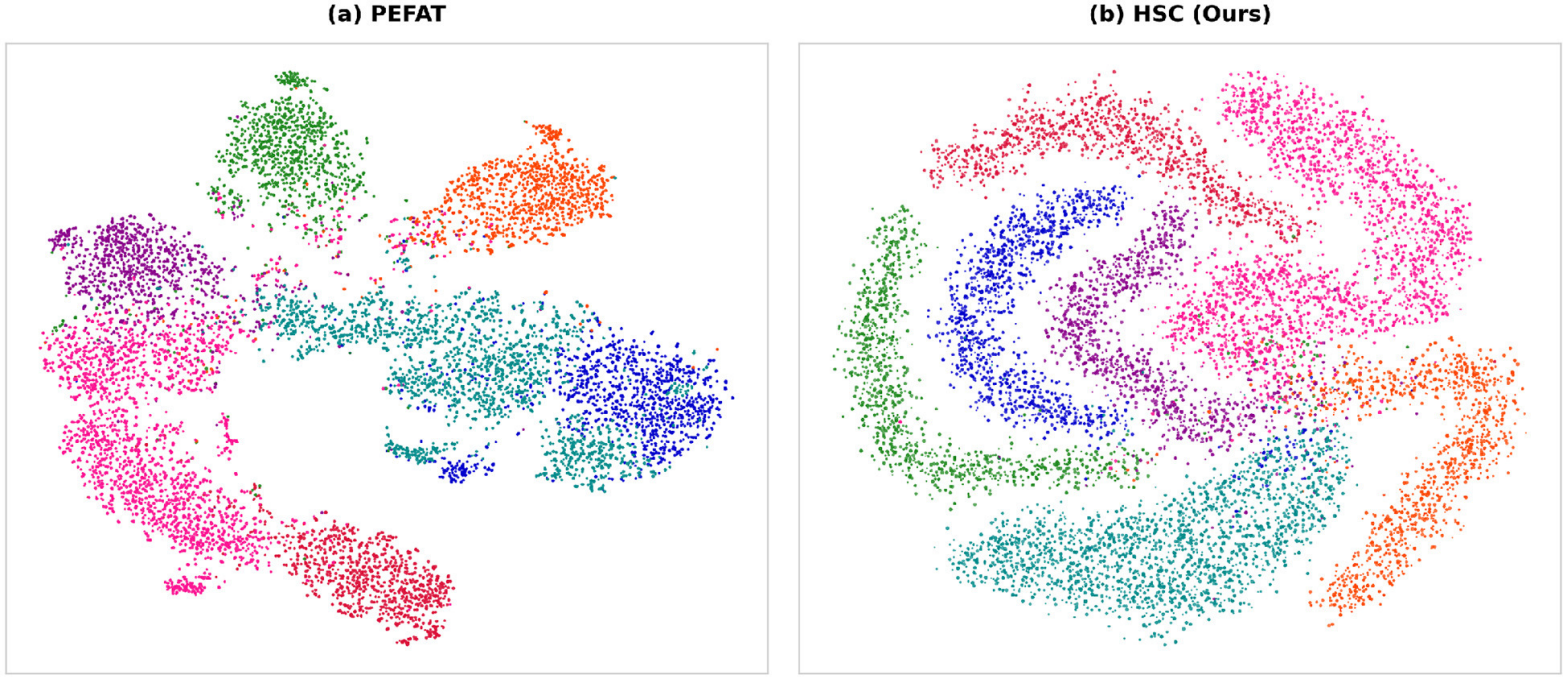
Comparison of learned feature representations via t-SNE projection. **(a)** Baseline method PEFAT; **(b)** proposed HSC method with dual contextual learning. The proposed method achieves better class separation and clustering.

To better understand the discriminative capability of learned representations, we visualize the feature distributions using t-SNE visualization. Figure 5 presents the 2D embeddings of test samples from ISIC 2018 dataset.

The baseline method (Figure 5a) shows relatively overlapped distributions with unclear boundaries between different skin lesion categories, indicating limited discriminative power. In contrast, our proposed HSC method (Figure 5b) exhibits more compact and well-separated clusters for each category, demonstrating superior inter-class separability and intra-class cohesion. The clearer decision boundaries and reduced feature confusion between different lesion types validate the effectiveness of our dual contextual learning mechanism.

#### 4.6.3 Confusion matrix analysis

**Figure 6:**
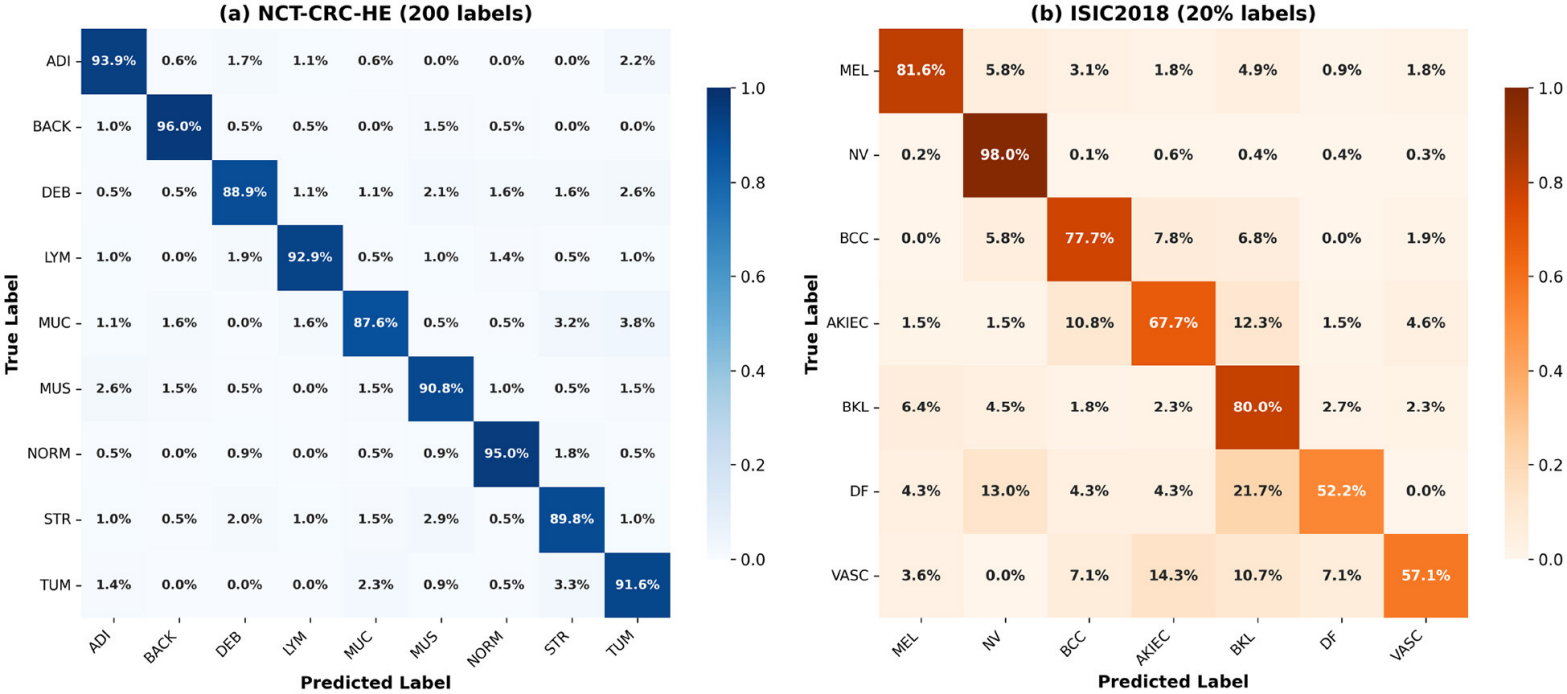
Confusion matrices of our HSC method on **(a)** NCT-CRC-HE with 200 labeled samples and **(b)** ISIC2018 with 20% labeled data. Each cell value represents the proportion of samples from the true class (row) predicted as the corresponding class (column). Diagonal values indicate per-class recall rates, while off-diagonal values reflect misclassification patterns.

Figure 6 shows the normalized confusion matrices for both datasets. On NCT-CRC-HE, most tissue categories achieve recall above 0.90, with BACK (0.96) and NORM (0.95) performing best. The main confusions occur between histologically similar tissues such as NORM-MUS and TUM-STR pairs. On ISIC2018, the class imbalance challenge is evident: NV achieves 0.98 recall while minority classes DF (0.55) and VASC (0.60) show lower performance. MEL is primarily confused with NV and BKL due to visual similarity. Despite this, our method maintains 0.82 MEL recall, demonstrating effective sensitivity preservation for clinically critical categories.

### 4.7 Computational complexity analysis

As shown in Table 6, we evaluate the computational overhead of our proposed HSC framework against the baseline. The theoretical training computation increases marginally from 8.64 to 8.82 GFLOPs, and the empirical training time per epoch only increases by approximately 3.1% (1.031×). This is attributed to the fact that our newly introduced HSC modules. However, given the substantial performance improvements, this sub-millisecond computational overhead is highly acceptable.

**Table 6:** Comparison of overall computational complexity (Training GFLOPs) and empirical training time.

Method	Total GFLOPs	Relative Time
PEFAT (Baseline)	8.64	1.000×
**HSC (Ours)**	**8.82**	**1.031×**

### 4.8 Empirical analysis of pseudo-label quality

**Figure 7:**
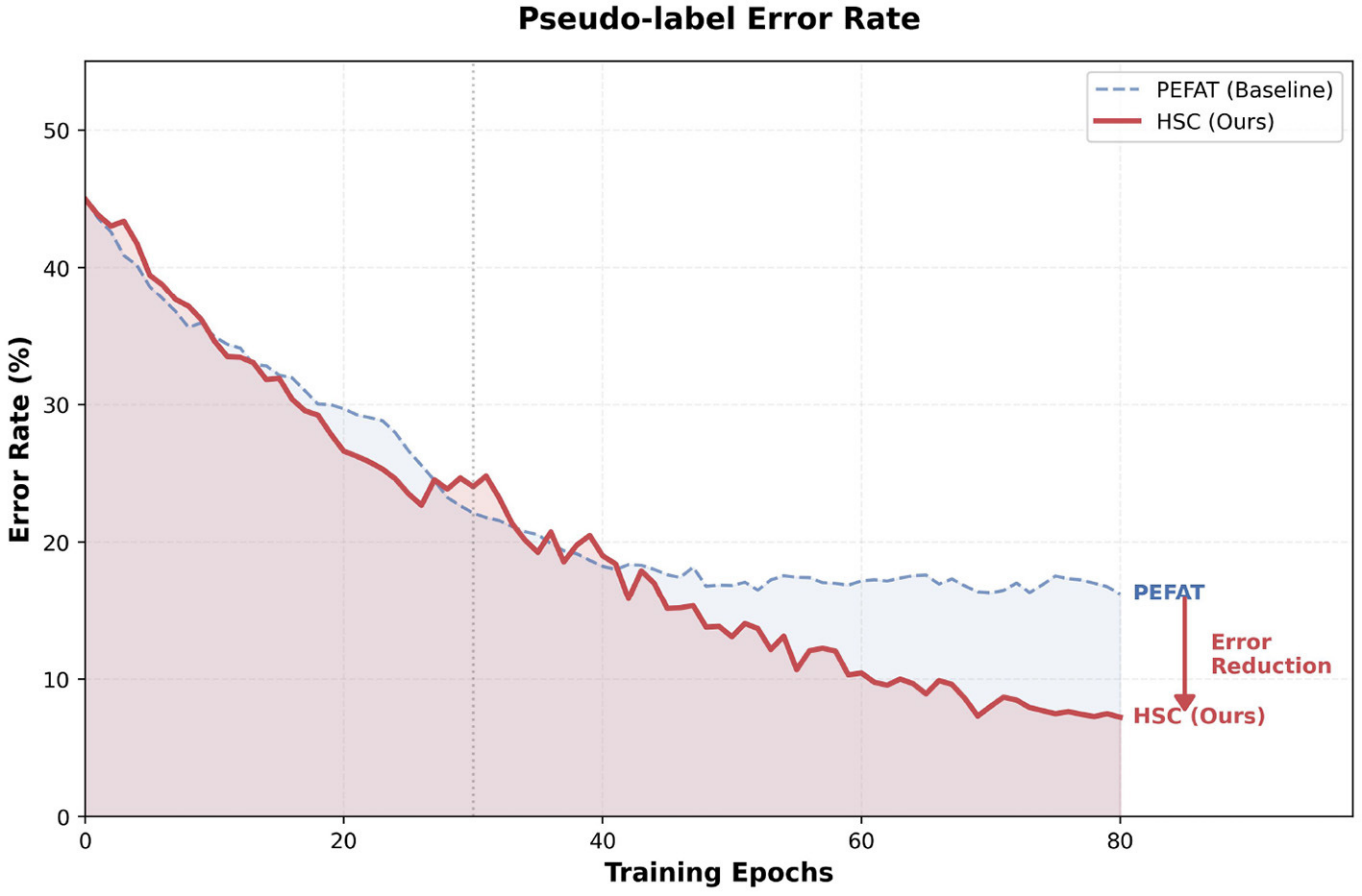
Pseudo-label error rate. Initially, HSC and PEFAT exhibit comparable quality during feature exploration. In later stages, the enhanced model capability driven by Dual Contextual Learning facilitates superior pseudo-label rectification and convergence.

To further investigate the effectiveness of the proposed HSC in mitigating confirmation bias, we monitor the evolution of pseudo-label error rates on the NCT-CRC-HE dataset (200 labeled data). As depicted in Figure 7, the error rates of both HSC and PEFAT are intertwined during the initial phase as the model focuses on fundamental feature exploration and discriminative learning. As training progresses, our Dual Contextual Learning effectively captures hierarchical semantic consistencies, which significantly refines the feature space and enhances the model's discriminative capacity. This improved capability creates a positive feedback loop that drives the generation of higher-fidelity pseudo-labels. Consequently, HSC achieves a substantial error reduction compared to the baseline, eventually converging at a superior error rate of approximately.

## 5 Conclusion

In this study, we propose Hierarchical Semantic Calibration (HSC), a novel semi-supervised learning framework for medical image classification. The core of our approach lies in the introduction of dual-level contextual calibration, which comprehensively exploits the semantic structure of medical images through two complementary mechanisms: local semantic neighborhood alignment that preserves fine-grained instance relationships, and global cluster-prototype consistency that captures broader category distributions. This hierarchical design effectively addresses the critical limitations of conventional pseudo-label refinement methods, which often fail to leverage the rich structural information embedded in unlabeled data. Our extensive experiments on ISIC 2018 and NCT-CRC-HE datasets demonstrate that HSC consistently outperforms state-of-the-art methods under limited supervision.

## Data Availability

The original contributions presented in the study are included in the article/supplementary material, further inquiries can be directed to the corresponding authors.
